# 3D cell culture model: From ground experiment to microgravity study

**DOI:** 10.3389/fbioe.2023.1136583

**Published:** 2023-03-24

**Authors:** Chiyuan Ma, Xianglong Duan, Xiaohua Lei

**Affiliations:** ^1^ Center for Energy Metabolism and Reproduction, Shenzhen Institute of Advanced Technology, Chinese Academy of Sciences, Shenzhen, China; ^2^ Institute of Medical Research, Northwestern Polytechnical University, Xi’an, China; ^3^ Second Department of General Surgery, Shaanxi Provincial People’s Hospital, Xi’an, China

**Keywords:** 3D culture, microgravity, hydrogel, tissue formation, dysfunction and regeneration

## Abstract

Microgravity has been shown to induce many changes in cell growth and differentiation due to offloading the gravitational strain normally exerted on cells. Although many studies have used two-dimensional (2D) cell culture systems to investigate the effects of microgravity on cell growth, three-dimensional (3D) culture scaffolds can offer more direct indications of the modified cell response to microgravity-related dysregulations compared to 2D culture methods. Thus, knowledge of 3D cell culture is essential for better understanding the *in vivo* tissue function and physiological response under microgravity conditions. This review discusses the advances in 2D and 3D cell culture studies, particularly emphasizing the role of hydrogels, which can provide cells with a mimic *in vivo* environment to collect a more natural response. We also summarized recent studies about cell growth and differentiation under real microgravity or simulated microgravity conditions using ground-based equipment. Finally, we anticipate that hydrogel-based 3D culture models will play an essential role in constructing organoids, discovering the causes of microgravity-dependent molecular and cellular changes, improving space tissue regeneration, and developing innovative therapeutic strategies. Future research into the 3D culture in microgravity conditions could lead to valuable therapeutic applications in health and pharmaceuticals.

## 1 Introduction

For a long time, two-dimensional (2D) cell culture has been used to study the physiological activities of cells in the complex human body using readily available flat plastic dishes. In a 2D culture system, the cells spread on flat and hard surfaces and proliferate unnaturally. As a result, their cellular morphology, functions, and overall behavior differ from those in the natural environment (Xu et al., 2000). Cells in the natural environment are embedded in the extracellular matrix (ECM), a fibrous three-dimensional (3D) structure, which ought to be viewed as a natural hydrogel (Schaefer and Schaefer, 2010). It is now possible to create these hydrogels that replicate ECM *in vitro* by using well-defined biopolymer models (collagen and fibrin) and other synthetic polymer models (Grinnell and Petroll, 2010; Liu et al., 2019). From an architectural perspective, the fibrous network of hydrogels with relatively large (on the order of millimeter size) holes makes it easy for cell growth, spreading, and metabolic chemicals to move between cells (Kikuchi et al., 2017). Because of these properties, hydrogels have been widely used in tissue engineering (Hou et al., 2022; Tarsitano et al., 2022).

Gravity impacts both physical and biological events on earth, influencing the development, equilibrium, and evolution of living systems. Reduced gravitational forces in space (microgravity: 10^–3^∼10^–4^ g) cause physiological changes in the human body, mainly in weight-bearing structures (White and Averner, 2001). The combined reactions to such changes and the re-adaptation of the human body during spaceflight and again upon re-entry pose substantial health dangers to space explorers. Space medicine research in the 21st century has tackled these health problems to better grasp the unknown mechanisms behind physiological alterations. Non-etheless, the medical curiosity about how these changes are organized extends beyond human space missions (Hides et al., 2017), calling for more extensive research in microgravity and countermeasure programs.

Cells were typically 2D exposed to real microgravity (RMG) in space or simulated microgravity (SMG) generated by a random positioning machine (RPM), which is a clinostat or a rotating wall vessel (RWV) bioreactor to investigate the effects of microgravity on cell growth (Briegleb, 1992; Goodwin et al., 1992). However, as previously stated, 2D-culture models are challenging to imitate real tissue. Thus, biotechnology and engineering advancements have permitted deploying more complex equipment and hydrogel-based 3D system for ground-based microgravity research. A fundamental understanding of biological alterations under microgravity conditions is critical not only for supporting human presence in space exploration but also for drug development and the potential development of novel tissue engineering and regenerative medicine.

This review focuses on the advances in cell growth and development achieved by culturing cells 2D and 3D under microgravity, particularly emphasizing the role of hydrogels-based 3D cell models. We anticipate that hydrogel-based 3D culture models will play an essential role in constructing organoids, discovering the causes of microgravity-dependent molecular and cellular changes, improving space medicine, and developing innovative therapeutic strategies.

## 2 3D vs. traditional 2D cell culture: Strategy and advantage

The tissue’s function is determined by cellular and non-cellular components (Nerger and Nelson, 2020). The more accurately a cell culture system can replicate such settings, the better cells will mimic the behaviors and reactions of cells *in vivo*. This is why 3D cell cultures are intriguing. 3D cell culture takes another step toward keeping cells alive, growing, and behaving as they do *in vivo* by focusing on simulating natural cell-matrix and cell-cell interactions (Park et al., 2021). 3D cell culture, in conjunction with a biomaterials-based scaffold, provides researchers with an unequaled capacity to replicate physiological compositions and spatial arrangements of cells *in vitro*.

Various 3D culture methods use biomaterials to increase the efficiency of culture and cell activities in various forms, such as hydrogels, solid scaffolds, decellularized natural tissue, and ultra-low attachment (ULA) surfaces (Tibbitt and Anseth, 2009; Fan et al., 2020). Knowledge of 3D culture techniques has grown dramatically, resulting in the creation of several applications. For example, hydrogels have similar qualities to natural ECMs, such as biocompatibility, biodegradability, and adjustable properties (such as shape, gel state, and mechanical strength) (Hwang et al., 2009; Lei et al., 2011a; Lei et al., 2011b; Smeriglio et al., 2015; Zhou et al., 2019; Wang et al., 2021a; Fournier and Harrison, 2021; Ma et al., 2021; Okita et al., 2021). In recent years, hydrogels have gotten much interest in tissue engineering research, and they are the 3D culture materials we will primarily discuss.

Hydrogels comprise natural, synthetic, and semi-synthetic polymers. Natural hydrogels are composed of natural components such as collagen, alginate, hyaluronic acid (HA), and others that support numerous biological activities by including various endogenous elements that can improve the survival, proliferation, and differentiation of many cell types (Lou et al., 2018; Jose et al., 2020). Synthetic hydrogels are made up of artificial molecules such as polyvinyl alcohol (PVA), poly-2-hydroxyethyl methacrylate (pHEMA), polyethylene glycol (PEG), and polyisocyanopeptide (PIC), which can give mechanical support to various cell types. At the same time, they are physiologically inactive and lack endogenous components (Zhu, 2010; Wang et al., 2021b). Thus, synthetic hydrogels must be modified with appropriate biological components to increase cellular function signals. Recently, it was reported that the combination of arginine-glycine-aspartic acid (RGD) groups with PIC-supported human umbilical vein endothelial cells (HUVECs) spread and formed an endothelial cell network, which is similar to the tissue form *in vivo* for 3D cell culture (Ma et al., 2022).

### 2.1 Improved cells viability and survival after long-term 3D culture

A long-term 3D organoid culture system was established for mouse and human primary hepatocytes by Matrigel, which is a hydrogel based on solubilized basement membrane preparation extracted from Engelbreth-Holm-Swarm (EHS) mouse sarcoma, a tumor rich in ECM proteins (Kleinman et al., 1982). In this system, single hepatocytes can create organoids that can be cultivated for several months while keeping important morphological, functional, and gene expression characteristics (Hu et al., 2018). Furthermore, PEG hydrogels were employed to culture and expand a range of neuronal and glial cell types (Lampe et al., 2010) and hepatocytes (Christoffersson et al., 2019) by simply changing the material properties of the hydrogel. In addition, RGD-modified alginate hydrogels accelerated the development of retinal pigment epithelium and neuroretina in 3D-cultured human embryonic stem cells (hESCs)/human induced pluripotent stem cells (hiPSCs) (Hunt et al., 2017).

### 2.2 Improved the efficiency of stem cell differentiation into specific cells

Since collagen is the most abundant natural type of fibrin hydrogel *in vivo* (Faraj et al., 2007), research has shown that collagen type II scaffolds may significantly improve the chondrogenic development of human mesenchymal stem cells (hMSCs) when compared to collagen type I hydrogel scaffolds (Tamaddon et al., 2017). Although type I and type II collagens support chondrogenic phenotypes in various ways, collagen hydrogel scaffolds can construct cartilage tissue (Tamaddon et al., 2017). For example, in a cartilage deficiency rat model, the collagen hydrogel scaffolds covered with rat mesenchymal stem cells (rMSCs) promoted rMSC chondrogenic development and had a statistically greater cartilage healing capability (Rouwkema and Khademhosseini, 2016). In addition, HA-based hydrogel scaffolds might stimulate the neural development of human-induced pluripotent stem cell-derived neural progenitors (hiPSC-NPCS) and the proliferation of neuroblastoma cells (Seidlits et al., 2010; Lei et al., 2011b; Yang et al., 2016). Another work encapsulated hiPSCs in HA-rich core-shell hydrogel microcapsules *via* microencapsulation to increase cell bulk and promote effective cardiac differentiation (Xu et al., 2021). In addition, growth factor-containing PVA hydrogels may accelerate the differentiation of mouse spermatogonial stem cells (mSCCs) into meiotic and postmeiotic cells (Kashani et al., 2020).

### 2.3 3D structures formation in a hydrogel-based cell culture

**FIGURE 1:**
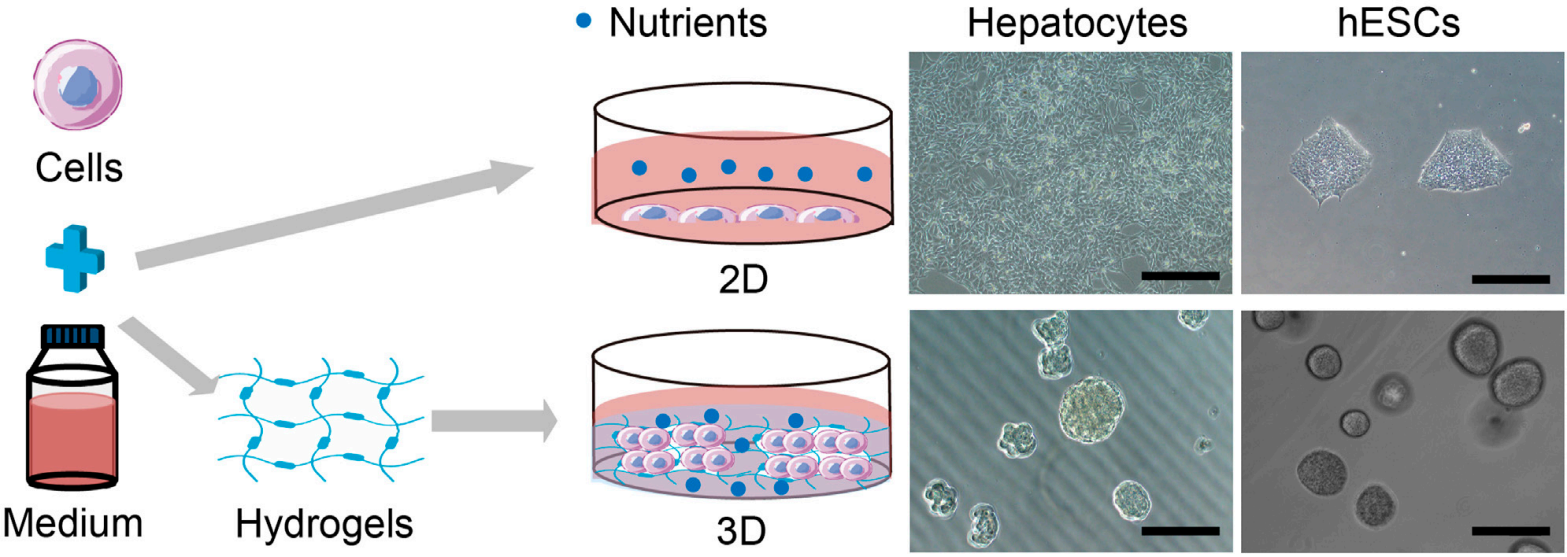
Comparison of hydrogel-based 3D cell culture and traditional 2D culture (Bar = 200 μm).

In a hydrogel-based 3D cell culture, cells can naturally form 3D structures rather than being restricted to a 2D surface. As shown in Figure 1, hepatocytes and hESCs form spheroids in PIC-based hydrogel (Bar = 200 μm). Similarly, the 3D culture experiments of chondrocytes showed that the collagen type I hydrogel scaffold could retain the chondrogenic phenotype of rat chondrocytes in a 3D growth pattern (Smeriglio et al., 2015; Jin and Kim, 2017; Kashani et al., 2020). Customizable HA hydrogels were also created with variable hardness for 3D rMSC growth (Wu et al., 2017). HA hydrogel scaffold can maintain the survival of bone marrow stromal cells (BMSCS) and promote direct tubular chondrogenic development (Ren et al., 2021). Furthermore, in 3D cultures of PVA hydrogel-coated cell plates, various human glioma cell lines (LN299, U87MG, and Gli36) may form tumor spheres like their morphology *in vivo* (Molyneaux et al., 2021). The alginate-collagen hydrogels improve cell adhesion of hiPSCs-derived neurons and stimulate the creation of complex neural networks in 3D culture models (Moxon et al., 2019).

The digested 2D cultured cells develop into a 3D cell mass after adding hydrogel into the cell suspension as a cell scaffold, as opposed to 2D culture with a flat morphology (Figure 1, left panel). Two types of cells, hepatocytes and hESCs, are depicted to show the difference between 2D patterned cells and 3D hydrogel-based cells (Figure 1, right panel). The hydrogel used here is polyisocyanopeptide (PIC) -based synthetic fiber hydrogels. hESCs, human embryonic stem cells; Bar = 200 μm.

### 2.4 Hydrogel-based 3D culture for the study of ECM on cell organization and function

It is known that a variety of growth factors may bind to ECM proteins. These secluded growth factors can produce gradients in concentration that direct the differentiation and morphogenesis of stem cells during *in vivo* development (Muncie and Weaver, 2018). Traditional 2D cell culture employs a liquid medium that does not allow for the storage and release of growth agents or the creation of concentration gradients. In contrast, 3D culture allows cells to construct 3D structures instead of being limited to a single layer in 2D (Jose et al., 2020). While more technically difficult than traditional 2D cell culture, hydrogel-based 3D cell culture facilitates normal cell-cell and cell-matrix interactions and tissue-specific activity. Materigel-based 3D cell culture, but not 2D models, increased tauopathy in human stem cell-derived neurons with familial Alzheimer’s disease by increasing the accumulation of β-amyloid aggregates in the ECM (Choi et al., 2014). PIC hydrogels enable the generation of mammary gland organoids from mammary fragments or pure single mammary epithelial cells by decorating with the adhesive peptide RGD for cell adhesion. Furthermore, the cell-gel interactions *via* the cell binding peptide density regulate the ratio of the major cell types in the mammary gland organoids (Zhang et al., 2020).

Given that alterations in cell-to-cell interactions and interactions with the ECM are the main effects of microgravity on cells and may alter cell fate through these effects (Andreeva et al., 2022), hydrogel-based 3D cell models should be employed for microgravity studies to study the response of human tissues and organs to microgravity.

## 3 Advance of cell growth and differentiation in microgravity

The study of the bio-effects of SMG and RMG on cell growth and differentiation is a current subject in space medicine, contributing to the applications of biomedical sciences on earth by using technologies designed to simulate microgravity (Lei et al., 2011a; Herranz et al., 2013; Pietsch et al., 2013). The absence of gravity affects the creation of proteins, apoptosis, proliferation, differentiation, migration, adhesion, and other cellular changes (Pietsch et al., 2011a). In the following, we will discuss the effects of microgravity on cell proliferation, differentiation, morphology, and adhesion in general, and advanced researches about those topics are listed in Table 1. Firstly, Cell proliferation, cell cycle, and programmed cell death (apoptosis) are the three primary indicators of how microgravity affects cell growth. Different cell types differ in these aspects under microgravity. For example, the proliferation of murine osteoblasts was enhanced by 6–21 days of culture on RPM and the expression of osteogenic marker genes in osteogenic (Braveboy-Wagner et al., 2021). RMG resulted in enhanced proliferation and a shortened cell cycle of neural stem cells within 38 days of culture in space (Shaka et al., 2022). The amount of Ki67-positive cells and formation of canning epithelium could be observed after 10 days of culture in human epidermal stem cells (hEpSCs) on RCCS (Lei et al., 2011a). However, human promyelocytic leukemic HL-60 cells showed a significant decrease in cell proliferation and expression of proliferating cell nuclear antigen (PCNA) and phosphorylated ERK1/2 and AKT proteins under SMG. Moreover, SMG increased DNA damage, apoptosis, and ROS formation (Singh et al., 2021).

**TABLE 1:** Summary of advanced research on cell growth and differentiation under microgravity (Last 5 years).

Microgravity condition	Cell line	Organ/issue	Device and exposure duration	Findings in microgravity (ug)	Reference
SMG	7F2 murine osteoblasts	Bone	RPM; Short-term experiments: 6 days. Long-term experiments: 21 days	SMG enhanced cell proliferation and expression of osteogenic marker genes in an osteogenic medium, even with the inhibitory effects on ALP activity	Braveboy-Wagner et al. (2021)
	hESCs	Human pluripotent stem cells	RPM; Short-term experiments: 2–4 days. Long-term experiments: 9 days	SMG facilitates hESC differentiation to HSPC with more efficient induction of CD34^+^CD31^+^ HEPs on day 4 and CD34^+^CD43^+^ HSPC on day 7, and these cells show an increased generation of functional hematopoietic cells in the colony-forming unit assay	Ma et al. (2021)
	Human promyelocytic leukemic HL-60 cells	Hepatocyte carcinoma	RCCS, 72 h	SMG decreased cell proliferation and expression of PCNA and phosphorylated ERK1/2 and AKT proteins. SMG increased the DNA damage, apoptosis, and ROS formation	Singh et al. (2021)
	Retinal pigment epithelial cells, ARPE19 cells	Visual system	3D clinostat	Actin cytoskeleton regulators were modulated in microgravity-stimulated ARPE19 cells. Excessive VEGF production and EMT marker expression also increased	Son et al. (2022)
	ADSCs	Human adipose-derived stem cells	FRC 6, 24, and 48 h; Scaffold-free; 1 g samples	Enhanced neural differentiation in neurons and increased neurotrophin expression and their specific Trk receptors, especially BDNF and TrkB	Zarrinpour et al. (2017)
	SCAP	Postnatal stem cells from the apical papilla of teeth	HARV 7 days; Sell spheroids were generated from SCAP through microwell-mediated self-condensation; NGF, EGF, and bFGF	3D nerve tissue formation	Kim et al. (2017)
	Nthy-ori 3-1	Thyroid tissue	RPM, 72 h; 1 g samples	The spheroid formation, AD cells; Cytokines are involved in the initiation of MCS formation through focal adhesion proteins	Warnke et al. (2017)
	Limbal fibroblasts (LFs)	Eye, corneal limbus; LFs have been shown to possess MSC characteristics	RCCS/HARV 3 days; 1 g controls	Greater number of MSC-like LF for stem cell therapy in ocular surface reconstruction; LF cells could differentiate into adipocytes, osteocytes, and chondrocytes	Pao et al. (2017)
	EPCs	PBMNC	3D clinostat	Most significant increase in CD34^+^ and double positive Dil-Ac-LDL-FITC-Ulex-Lectin cells, both EPC markers. Enhancing the number and angiogenic potential of EPCs	Wang et al. (2019)
	HepG2Human biliary tree stem/progenitor cells (hBTSCs)	Hepatocyte carcinoma	RCCS	SMG promotes the formation of 3D cultures and stimulates pluripotency and glycolytic metabolism in human hepatic and biliary tree stem/progenitor cells	Costantini et al. (2019)
	Human epidermal stem cells (hEpSCs)	Epidermis-like structure	RWV bioreactor, Cytodex-3 microcarriers	hEpSCs aggregated on the microcarriers and formed multilayer 3D epidermis structures	Lei et al. (2011a)
	ADSCs	Adipose tissue	2Dclinostat, CTGF	Differentiation to fibroblast cells. Col1 and Collll, MMP1, ITGB1, and FSP1 gene expression changes involved	Ebnerasuly et al. (2017)
	HCT116	Colorectal cancer	RCCS	CSC; CD133/CD44 dual positive cells, giant cancer cells with complete nuclear localization of YAP	Arun et al. (2017), Arun et al. (2019)
	Rabbit ADSCs and bone marrow stromal cells	Cartilage	RCCS, a novel cell carrier derived from natural cartilage ECM	improved the induction of stem cell chondrogenesis as well as *in vivo* repair of cartilage lesions in a rabbit model	Yin et al. (2018)
RMG	NSCs	Central nervous	Spaceflight with SpX-21 aboard the ISS for 39.6 days	RMG resulted in enhanced proliferation, a shortened cell cycle, and a larger cell diameter of NSCs; Frequent occurrence of ACD, including incomplete cell division, where cytokinesis is not successfully completed, and multi-daughter cell division of NSCs	Shaka et al. (2022)
	HMVEC	Vessels	Spaceflight to ISS with SpaceX CRS-8: 5 days (7 days) and 12 days (14 days) μg; RPM 7 days	Tubular structures, spheroids. AD cells	Pietsch et al. (2017)
	Pluripotent stem cell-derived cardiomyocytes	Heart	ISS	Alterations in hiPSC-CM calcium handling showed 2635 differentially expressed genes	Wnorowski et al. (2019)
	CPC	Cardiac tissue	ISS, 2D Clinostat	Hippo signaling; upregulation of downstream genes: YAP1 and SOD2	Camberos et al. (2019)
	Adult and neonatal CPCs	Cardiac tissue	ISS	Only neonatal CPCs showed an increased expression of early developmental markers and an enhanced proliferative potential	Baio et al. (2018)
	hiPSC-derived cardiac progenitors	Cardiac tissue	ISS, 3 weeks	Microgravity cultures had 3-fold larger sphere sizes, 20-fold higher nuclei counts, and increased expression of proliferation markers. Improved Ca2^+^ handling and increased expression of contraction-associated genes. Short-term exposure (3 days) of cardiac progenitors to space microgravity upregulated genes involved in cell proliferation, survival, cardiac differentiation, and contraction	Rampoldi et al. (2022)
	hBMSCs	Bone marrow	ISS	SMG stresses reverting to a quiescent state	Bradamante et al. (2018)

Abbreviations: SMG, simulated microgravity; RMG, real microgravity; ALP, alkaline phosphatase; hESCs, human embryonic stem cells; HEPs, hemogenic endothelium progenitors; HSPC, hematopoietic stem/progenitor cell; NSCs, neural stem cells; ACD, abnormal cell division; AD, adherent cells; EGF, epidemal growth factor; MCS, multicellular spheroids; RCCS, rotary cell culture system; RPM, random positioning machine; RWV, rotating wall vessel; ISS, international space station; ROS, reactive oxygen species; PCNA, proliferating cell nuclear antigen; ERK 1/2, extracellular signal-regulated kinase 1/2; ROS, reactive oxygen species; ADSCs, adipose derived stem cells; bFGF, basic fibroblast growth factor; BMSC, bone mesenchymal stem cell; CPCs, cardiac progenitor cells; EPC, endothelial progenitor cell; FRC, fast rotating clinostat; HARV, high-aspect rotating vessel; NGF, nerve growth factor; SCAP, stem cell from apical papilla; CSC, cancer stem cell; EMT, epithelial-mesenchymal transition; HMVEC, human microvascular endothelial cell; TGF, transforming growth factor; Col1, collagen type I gene; ColIII, collagen type III gene; ESCs, embryonic stem cells; EPCs, endothelial progenitor cells; FSP1, fibroblast-specific protein 1 gene; hBTSCs, human biliary tree stem/progenitor cells; MMP1, matrix metalloproteinase 1 gene; SOD2, superoxide dismutase 2; YAP1, yes-associated protein 1 gene.

### 3.1 Effect of microgravity on differentiation of stem cells

Most studies show that microgravity affects cell differentiation by promoting reagent-induced differentiation of stem cells or specific cell types into specific tissue types. Short-term exposure (3 days) of cardiac progenitors to space microgravity upregulated genes involved in cardiac differentiation (Rampoldi et al., 2022). hBMSCs were affected by RMG and responded to RMG stresses, reverting to quiescence after a moderate osteogenic differentiation aboard ISS for 3 weeks (Bradamante et al., 2018). It was demonstrated that SMG facilitates hESCs to differentiate into hematopoietic stem cells (HSCs) and progenitor cells with more efficient induction of CD34^+^ CD31^+^ hemogenic endothelium progenitors (Ma et al., 2021). ADSCs displayed enhanced neural differentiation in neurons and increased neurotrophin expression and their specific Trk receptors, especially BDNF and TrkB, when cultured on fast rotating clinostat (Zarrinpour et al., 2017). When Postnatal stem cells from the apical papilla of teeth were cultured with NGF, EGF, and bFGF on high-aspect rotating vessels for 7 days, they formed nerve tissue (Kim et al., 2017). Limbal fibroblasts (LFs) cells could differentiate into adipocytes, osteocytes, and chondrocytes on RCCS/HARV for 3 days, compared to 1 g controls (Pao et al., 2017). Neonatal cardiac progenitor cells showed an increased expression of early developmental markers on ISS, 3 weeks (Baio et al., 2018). This less visible differentiation could be attributed to additional environmental factors or the poor cultural conditions of space travel experiments. However, some evidence suggests that microgravity can preserve pluripotency. Mouse ESCs can be maintained without leukemia inhibitory factor (LIF) and retain pluripotency under a simulated microgravity environment (Kawahara et al., 2009). Similar results were obtained for cancer stem cells (CSCs), which have similar differentiation potential to ESCs (Arun et al., 2017). SMG also increased stemness in human colorectal cancer cell HCT116 using RCCS, indicating CD133/CD44 dual-positive cells (Arun et al., 2019).

### 3.2 Effect of microgravity on cell adhesion, morphology, and cytoskeleton

Another important aspect of the effect of microgravity on cells is the influence on cell adhesion, morphology, and cytoskeleton. When subjected to microgravity, some cells grew into a monolayer altering their growth behavior, while the remaining continued to develop naturally (Infanger et al., 2006; Ulbrich et al., 2010; Pietsch et al., 2011b). Although these cells detect the absence of gravity within seconds (Ulbrich et al., 2011; Grosse et al., 2012), it takes at least 12 h to see spheroids (Infanger et al., 2006; Pietsch et al., 2011b) and up to 7 days to see constructions like tubes resembling an intima floating in a culture flask (Grimm et al., 2009; Grimm et al., 2010). Our previous study found that SMG promotes hESCs to differentiate between HSCs and hematopoietic progenitor cells (HSPC). Interestingly, HSPC prefers floating growth, and transcriptome sequencing results showed that cell adhesion and ECM-related genes were downregulated (Ma et al., 2021). When retinal pigment epithelial cells, ARPE19 cells, were cultured on a 3D clinostat, actin cytoskeleton regulators were modulated, and the cells showed multilayered growth with increased expression of epithelial-mesenchymal transition (EMT) markers (Son et al., 2022).

Exposure of cells from epithelial tissue to microgravity creates 3D structures. For example, human microvascular endothelial cells (HMVEC) were exposed to RMG for 5 days and 12 days and SMG for 7 days, showing tubular structures (Pietsch et al., 2017). The yes-associated protein (YAP) and HIPPO signaling changes, known to be correlated with organ growth, cytoskeleton, and stress sensing, are also present in specific cells with enlarged morphology (Ma et al., 2019). For example, the upregulation of Hippo signaling with downstream genes, YAP1, was detected in cardiac progenitor cells (CPCs) when cultured on ISS and 2D clinostat (Camberos et al., 2019). Colorectal cancer cells, HCT116, are giant cancer cells formed with complete nuclear localization of YAP in the culture on RCCS (Arun et al., 2019). Changes in the cell adhesion, morphology, and cytoskeleton, which can impact various cellular functions, maybe a possible substrate for a cell’s response to microgravity. Since the 2D cell model differs from the *in vivo* stress environment, eliminating the effects of microgravity on cells from the experimental model should ensure that they are in a 3D development state akin to the *in vivo* environment.

## 4 Hydrogel-based 3D cell model for tissue formation, dysfunction, regeneration, and drug testing under microgravity conditions

**FIGURE 2:**
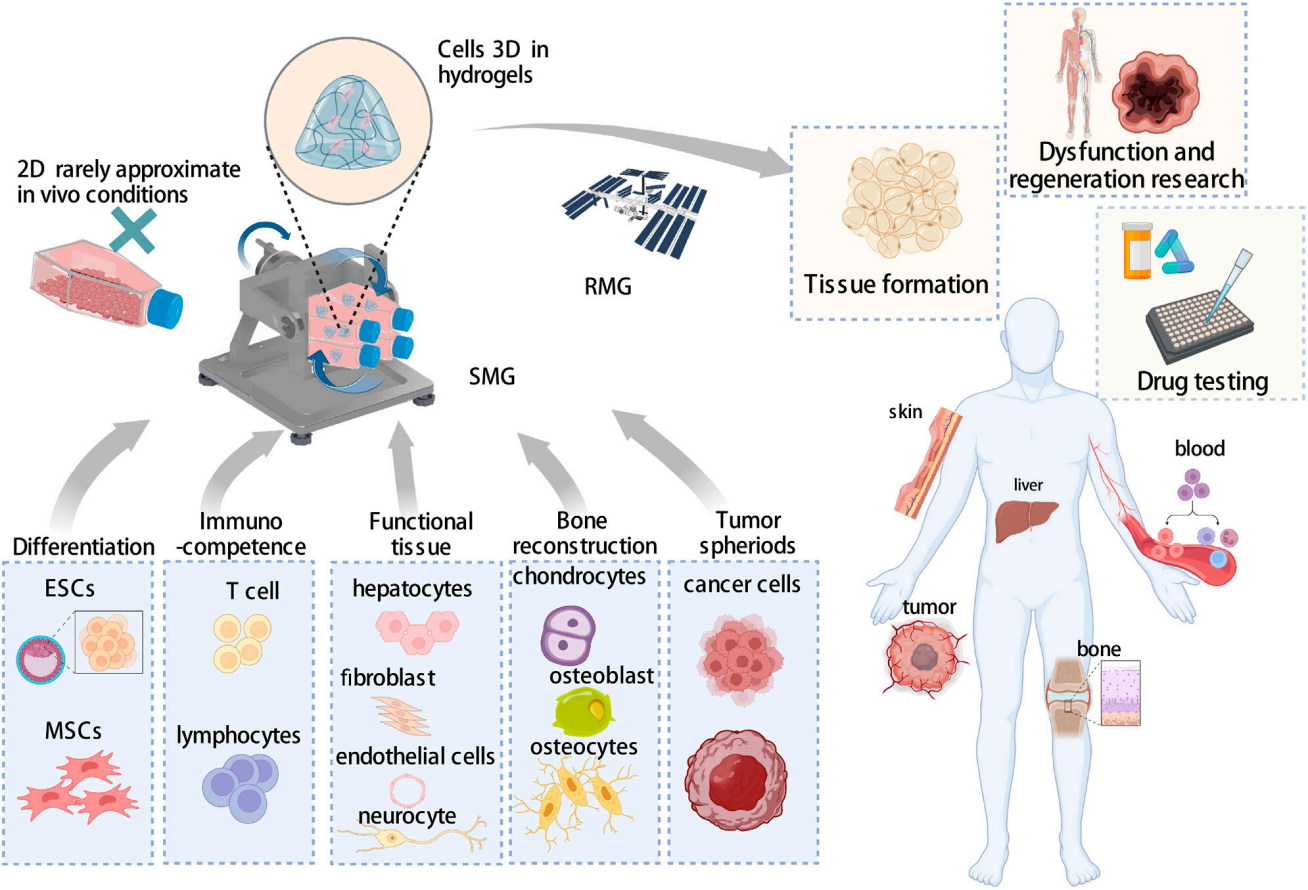
3D culture system with hydrogels for microgravity research.

In space circumstances, it has been discovered that there are some discrepancies in the biological effects between *in vivo* and *in vitro*, such as bone metabolism (Loomer, 2001), neuronal adaptation (Kohn and Ritzmann, 2018), skin health, and wound healing (Kasiviswanathan et al., 2020), which may be attributed to complex environments *in vivo*, as cell-cell contact. Given the obvious advantages of 3D culture methods, many studies have attempted to use 3D methods in microgravity research. Except for the scaffold-free clinostat for 3D culture (Aleshcheva et al., 2016), to better simulate the microenvironment of cell interaction *in vivo*, various biomaterials have been used as scaffolds for 3D culture, as a mixture of inorganic salt and collagen (Fournier and Harrison, 2021), matrix cell (Jackson et al., 2020) to study bio-effects of microgravity. As a result, these technologies constitute a novel paradigm for the organization of a wide range of tissues, including cartilage regeneration (Zhou et al., 2019), artificial vascular construction (Rouwkema and Khademhosseini, 2016), and generation of various organ tissues (Herranz et al., 2013; Luo et al., 2013; Zhang et al., 2014; Salerno-Goncalves et al., 2016) and cancer spheroids (Qian et al., 2008; Ulbrich et al., 2011; Ma et al., 2014). Furthermore, these aggregates are utilized to research the molecular pathways involved in angiogenesis (Rouwkema and Khademhosseini, 2016), osteogenesis (Braveboy-Wagner and Lelkes, 2022; Masini et al., 2022), cancer formation (Ma et al., 2014; Dietrichs et al., 2022), and pharmacological testing (Nishikawa et al., 2005) (Figure 2). The multiple advantages of the 3D culture of hydrogels are already discussed in Part 2. The specific applications of hydrogel-based 3D culture in microgravity are listed in Table 2.

**TABLE 2:** Natural, synthetic, and part hydrogels for 3D cell cultures applied in microgravity study.

Source of Hydrogels	Properties	Materials	Cells	Applications
Natural	Provide viscoelasticity and fiber equivalent to ECM; It has good biocompatibility; Endogenous factors can support cell activity	Collagen	Fibroblast Sapudom et al. (2021)	Alpha-smooth muscle actin (αSMA) expression and translocation of Smad2/3 into the cell nucleus were reduced, matrix remodeling and production were decreased under SMG
			T cells ElGindi et al. (2022)	
			NOR-P1 cells and fibroblasts or minced human pancreatic carcinoma Nakamura et al. (2002)	Resting and activated stages and the effects of SMG on the T cells transcriptome and nuclear irregularities
			Primary human lymphocytes, endothelial cells, and fibroblasts Salerno-Goncalves et al. (2016)	*In vitro* studies of complex pancreatic carcinoma tissue
		Alginate	mESCs Hwang et al. (2009)	Alginate encapsulation, and rotary microgravity bioreactor in bone tissue engineering
		Alginate and chitosan	Chondrocytes Zhou et al. (2019)	Design and construction of cartilage regeneration in future tissue engineering applications
			Rat normal liver cell BRL-3A Long et al. (2022)	The microgravity culture condition can enhance cell proliferation and promote the formation of silk fibroin
		Chitosan/gelatin	ADSC Zhu et al. (2017)	Promoted cellular proliferation and chondrogenic differentiation of ADSCs inside chitosan/gelatin hybrid hydrogel scaffolds
		Gelatin-alginate	Human cardiac AC16 cardiomyocytes, fibroblasts, and microvascular endothelial cells Alonzo et al. (2022)	3D bio-printed “cardiac organoid” exhibited contractile functions
		Matrigel	Human epidermal keratinocytes HaCaT Choi et al. (2021)	Increased the endothelial cell arrangement, decreased the diameter of keratinocytes and fibroblast co-cultured spheroids, representing skin thinning. Cytokeratin-10 expression was significantly increased, representing possible canceration
		Hyaluronic acid-based microcarriers	Human hepatocytes MSC (HCT116) Devarasetty et al. (2017)	3D organoids; formation of a stroma-like tissue surrounding the tumor foci and hepatocytes; less sensitive to fluorouracil drug treatment
Synthetic	Good mechanical strength, providing structural support for various cell types in three-dimensional cell culture	PLGA	Chondrocytes Emin et al. (2008)	Forming articular neo-cartilage tissue *in vitro*
			hPDLFs Inanc et al. (2006)	
			hDPSCs Li et al. (2017)	An effective method for the production of hPDLF-PLGA and hDPSCs-PLGA constructs with increased osteogenic and odontogenic differentiation potential
Part	ECM microenvironment characteristics and faster stress relaxation	Type I collagen and hydroxyapatite (collagen-HA)	Osteocytes Fournier and Harrison. (2021)	Allowed the osteocytes to survive and grow for up to 6 months
			hPDLFs Inanc et al. (2007)	Improved the osteogenic differentiation potential of hPDLFs
		Polystyrene and collagen microspheres	hASCs Mashiko et al. (2021)	hASCs expressed higher levels of pluripotent markers (OCT4, SOX2, NANOG, MYC, and KLF) and had improved abilities for proliferation, colony formation, network formation, and multiple-mesenchymal differentiation

### 4.1 Hydrogel-based 3D cell model for the study of tissue formation and drug testing

For tissue engineering, various hydrogels were combined with a microgravity bioreactor. A multicellular 3D organotypic model used collagen I matrix of the human intestinal mucosa was composed of an intestinal epithelial cell line and primary human lymphocytes, endothelial cells, and fibroblasts cultured under microgravity provided by the RWV bioreactor (Salerno-Goncalves et al., 2016). When chondrocytes were seeded onto poly (DL-lactic-co-glycolic acid) (PLGA) sponges and cultured in a chondrogenic induction medium containing TGF-β 1 for 3 weeks, the engineered cartilage then emerged in a microgravity bioreactor for another 3 weeks. The results showed that it had a similar structure and composition to native rat cartilage (Emin et al., 2008). Human periodontal ligament fibroblasts (hPDLFs) were 3D cultivated on a mineralized PLGA scaffold to mimic microgravity in the NASA-approved bioreactor. The outcomes demonstrated a successful strategy for producing hPDLF-PLGA structures with improved osteogenic potential using a 3D system and microgravity settings (Inanc et al., 2006). hPDLFs encapsulated in C/HA microspheres exhibited significantly higher osteogenic differentiation potential when compared to those not encapsulated. The 3D-osteogenic culture environment can potentially improve the osteogenic differentiation of hPDLFs (Inanc et al., 2007). The collagen-HA is a type of implant material used to create a permanent implant. The collagen-HA material allowed the embedded MLO-Y4 cells to survive and grow for 6 months. This technology creates permanent implants for patients with spinal cord injuries (Fournier and Harrison, 2021). However, microgravity raises the likelihood of irreversible changes that weaken skeletal integrity and the gradual start of fracture injuries in space travelers (Nelson et al., 2009; Genah et al., 2021). According to the findings of the previous studies, the 3D -matrix bone differentiation model can be used to test potential drugs against bone loss or to promote bone regeneration.

### 4.2 Hydrogel-based 3D cell model for stem cell differentiation and tissue regeneration

Hydrogel 3D culture models were used to investigate the influence of microgravity on stem cell development, an important research area in regenerative medicine and tissue engineering. An efficient and integrated 3D bioprocess has developed based on the encapsulation of undifferentiated mouse embryonic stem cells (mESCs) within alginate hydrogels. The osteogenic lineage’s morphological, phenotypic, and molecular characteristics were represented in 3D mineralized constructions with mechanical strength and mineralized calcium/phosphate deposition. This bioprocess represents a significant advance in bone differentiation from mESCs (Hwang et al., 2009). Similar findings have been discovered in improved odontogenic differentiation abilities of Human dental pulp stem cells (hDPSCs) on PLGA scaffolds in the 3D SMG culture system (Li et al., 2017), indicating that these models have the potential to be used to explore tissue regeneration processes in regenerative medicine and microgravity conditions.

It may be preferable to create a physiological and pathological research model similar to that used *in vivo*. Human pancreatic cancer NOR-P1 cells, fibroblasts, or minced pancreatic carcinoma tissue were grown in solid collagen gels for seven days in a microgravity environment. Compared to NOR-P1 3D cultures treated to the static 1 g condition, cultures subjected to the SMG condition had more mitotic, cycling (Ki67-positive), nuclear factor-kappa B-activating cells and fewer apoptotic cells. Additionally, compared to static culture conditions, human pancreatic cancer specimens better preserved the original carcinoma tissue’s heterogeneous makeup and cellular activity (measured by the cycling cell ratio and mitotic index) (Nakamura et al., 2002). When fibroblast differentiation was studied in SMG using collagen-based 3D matrices to approximate interstitial tissue, SMG exposure decreased alpha-smooth muscle actin (SMA) expression and Smad2/3 translocation into the cell nucleus compared to the 1 g control (Sapudom et al., 2021). Compared to 2D cell culture, 3D cell culture attenuates the effects of SMG on the T cells transcriptome and nuclear abnormalities, which were closer to the *in vivo* findings (ElGindi et al., 2022).

Compared to 2D culture, 3D cell culture is more effective for enhancing cell differentiation and organ-like tissue formation from a variety of cells to study the bio-effects of microgravity (Figure 2, lower panel). Exposing 3D cells to SMG circumstances increased the knowledge of biological response mechanisms to SMG and RMG in several tissues, including the liver, bone, vasculature, skin, and capillary tissue. Microgravity affects physiology, pathology, and medical research (Figure 2, right panel). MSCs, mesenchymal stem cells; ESCs, embryonic stem cells (the Figure was created with BioRender.com).

## 5 Conclusion

As the studies above suggest, microgravity research would provide insight into the basic mechanisms of tissue dysfunction and regeneration under SMG or RMG, which could be applied to terrestrial settings. Additionally, combining tissue engineering approaches with ground-based platforms will open up new avenues for space physiology and aging research and accelerate the creation of novel tissue-engineered constructions. This can be done by using cells from the patient’s body or cell lines. These platforms can be made from various materials, including biomaterials (especially hydrogels), plastics, and composites. However, most of the 3D tissue and organ models cultured in microgravity environment are composed of single-cell types, which cannot simulate the complete function of organs that are comprise of complex multi-cell types. Moreover, the research on 3D cell matrix and cell interaction is not deep enough. Adjusting the physical and chemical properties of materials may give appropriate mechanical feedback to cells, that may be more conducive to the construction of some organizational models. In conclusion, 3D culture holds great promise for the future of biological research in microgravity. Tissue engineering and microgravity interfacial research will drive breakthroughs in both fields in the coming years.
